# Supplementary material for: De-simplifying single-tablet antiretroviral treatments for cost savings in France: From the patient perspectives to a 6-month follow-up on generics
Source: PLoS One. 2020 Sep 25;15(9):e0239704. doi: 10.1371/journal.pone.0239704 (PMC7518587; doi:10.1371/journal.pone.0239704)
Supplement: S1 File — The survey is written in French and translated in English. (DOCX) [file pone.0239704.s001.docx]

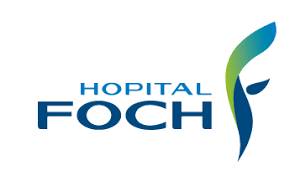
Enquête sur la prise de génériques d’antirétroviraux

Date de consultation :

Médecin : DZ🞏 CM🞏 EF 🞏

| Patient |
| --- |
| Initiales Nom : Initiales Prénom :  H / F Année de naissance :  Région de naissance : ASS 🞏 M(aghreb) 🞏 France 🞏 Autres 🞏  Catégorie socioprofessionnelle (INSEE) : 🞏 Agriculteur, exploitant 🞏 Ouvrier 🞏 Profession intermédiaire 🞏 Employé 🞏 Artisan, commerçant, chef d’entreprise 🞏 Cadre et profession intellectuelle supérieure 🞏 Etudiant 🞏 Inactif, sans emploi |
| Pathologie |
| Année diagnostic VIH :  Année début de traitement ARV :  VHB + : O / N Géno dispo O / N Résistances O / N |
| Traitements |
| Co-médications (au long cours) O / N  Traitement actuel : 🞏 Eviplera 🞏 Triumeq  Pharmacie où vous récupérez vos traitements :   - Rétrocession : 🞏 Foch 🞏 Autre établissement - Ville 🞏 |

**QUESTION 1 :**

A/ Avez-vous une idée du prix de votre traitement ? Oui / Non

B/ Combien par mois coûte-t-il d’après vous ? :

🞏 < 500 euros 🞏 500-1000 euros 🞏 1000-2000 euros 🞏 > 2000 euros

**QUESTION 2 :**

A/ Si vous aviez deux comprimés à prendre en même temps à la place d’un seul, cela vous ennuierait-il ?

Pas du tout 🞏 Un peu 🞏 Moyennement 🞏 Beaucoup 🞏

B/ Si vous aviez deux boites de médicament à la place d’une seule, cela vous ennuierait-il ?

Pas du tout 🞏 Un peu 🞏 Moyennement 🞏 Beaucoup 🞏

**QUESTION 3 :**

A / Avez-vous l’habitude de prendre des médicaments sous forme de génériques ? Oui / Non

B/ Si vous avez répondu non à la question A : Etes-vous de façon générale opposé à la prescription des médicaments génériques ? Oui / Non

**QUESTION 4 :**

D'une manière générale, avez-vous confiance dans les génériques ?

Pas du tout 🞏 Un peu 🞏 Moyennement 🞏 Beaucoup 🞏

**QUESTION 5**:

A/ Au cours du mois, avez-vous déjà oublié de prendre votre médicament ?

Jamais 🞏 Une fois par mois 🞏 Une fois par semaine 🞏 Plusieurs fois par semaine 🞏

B/ Depuis la dernière consultation, avez-vous été en panne de médicament? Oui / Non

**QUESTION 6 :**

Pensez-vous qu’il y ait plus de risques que vous preniez mal (oubli de prise, saut de prise,…) votre traitement si celui-ci est composé de 2 comprimés au lieu d’1 par jour ? Oui / Non

**QUESTION 7 :**

Etes-vous d’accord pour que votre traitement actuel soit remplacé par 2 cps à prendre en même temps dont l’un est un médicament générique (une seule réponse possible) ?

A/ OUI (tout de suite) 🞏

B/ Peut-être (plus tard, je dois réfléchir…) 🞏

C/ Selon l’avis du médecin référent 🞏

D/ NON 🞏

**QUESTION 8 :** Pour quelles raisons avez-vous répondu B, C ou D à la question 7 ? (plusieurs réponses possibles) :

A/ Préfère n’avoir qu’une seule boite de médicament 🞏

B/ Préfère ne prendre qu’un seul comprimé 🞏

C/ Peur d’avoir du mal à prendre plusieurs comprimés 🞏

D/ N’a pas confiance dans les génériques 🞏

E/ Prends déjà trop de comprimés 🞏

F/ Peur d’oublier de prendre un des deux comprimés 🞏

G/ Autre : ……………………………………………………… 🞏


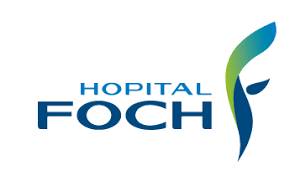
Survey on the use of generic antiretroviral drugs

Medical consultation’s date:

Physician: DZ🞏 CM🞏 EF 🞏

| Patient |
| --- |
| Initial of surname : Initial of first name :  Gender : Male / Female Year of Birth :  Birth Region: Sub-Saharan Africa 🞏 Maghreb 🞏 France 🞏 Other 🞏  Socio-professional Status: 🞏 Lower supervisory and technical occupations 🞏 Intermediate occupations (clerical, sales, service) 🞏 Lower managerial and professional occupations 🞏 Small employers and own-account workers 🞏 Higher managerial and professional occupations 🞏 Never worked or long-term unemployed |
| Disease |
| Year of HIV diagnosis :  Year of starting antiretroviral treatment:  HBV co-infection : Yes / No Viral Genotype determination : Yes / No History of genotypic resistance Yes / No |
| Treatments |
| Non-ARV daily comedications Yes / No  Actual ARV Therapy:  🞏 abacavir/lamivudine/dolutegravir (Triumeq®, Viih Healthcare)  🞏 emtricitabine/tenofovir disoproxil fumarate/rilpivirine (Eviplera®, Gilead Sciences)  ARV dispensing pharmacy:   - Hospital: 🞏 Ours 🞏 Another - Community : 🞏 |

**QUESTION 1 :**

A/ Do you have any idea how much your drug costs? Yes / No

B/ How much do you think it costs per month? :

🞏 < 500 euros 🞏 500-1000 euros 🞏 1000-2000 euros 🞏 > 2000 euros

**QUESTION 2 :**

A/ Would you mind having two tablets to take at the same time instead of one? Not at all 🞏 A little 🞏 Moderately 🞏 A lot 🞏

B/ Would you mind having two boxes of medication per month instead of one? Not at all 🞏 A little 🞏 Moderately 🞏 A lot 🞏

**QUESTION 3 :**

A / Are you used to take generic drugs? Yes / No

B/ If you answered no to question A: Are you generally opposed to the prescription of generic drugs? Yes / No

**QUESTION 4 :**

In general, do you trust generics? Not at all 🞏 A little 🞏 Moderately 🞏 Greatly 🞏

**QUESTION 5**:

A/ Have you ever forgotten to take your medication in the previous month?

Never 🞏 Once a month 🞏 Once a week🞏 Several times a week 🞏

B/ Since your last visit, have you ever run out of medicine? Yes / No

**QUESTION 6 :**

Do you think there is a greater risk that you will take your treatment incorrectly (forgetting to take it, skipping it...) if it consists of 2 tablets instead of 1 per day? Yes / No

**QUESTION 7 :**

Do you agree to switch from your current treatment to two tablets to be taken at the same time, one of which is a generic drug (only one possible response)? A/ Yes (right away) 🞏

B/ Maybe (Later, I have to think about it) 🞏

C/ Maybe (In discussion with the referring doctor) 🞏

D/ No 🞏

**QUESTION 8 :** For what reasons did you answer B, C or D to question 7? (multiple answers possible) : A/ Prefers to have only one box of medicine per month 🞏

B/ Prefers to take only one tablet per day 🞏

C/ Fear of having trouble taking multiple tablets 🞏

D/ Does not trust generic drugs 🞏

E/ Already taking too many pills 🞏

F/ Fear of forgetting to take one of the two tablets 🞏

G/ Others: ……………………………………………………… 🞏
