# Supplementary material for: De-simplifying single-tablet antiretroviral treatments for cost savings in France: From the patient perspectives to a 6-month follow-up on generics
Source: PLoS One. 2020 Sep 25;15(9):e0239704. doi: 10.1371/journal.pone.0239704 (PMC7518587; doi:10.1371/journal.pone.0239704)
Supplement: S3 File — The survey is written in French and translated in English. (DOCX) [file pone.0239704.s003.docx]

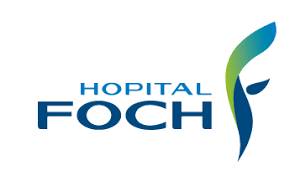
Enquête sur le suivi post-switch de génériques d’antirétroviraux

Date de consultation :

Médecin : DZ🞏 CM🞏 EF 🞏

| Patient |
| --- |
| Initiales Nom : Initiales Prénom :  H / F Année de naissance :  Depuis combien de temps avez-vous commencé le nouveau traitement ? ………………….. |

**QUESTION 1 :**

Etes-vous satisfait de votre traitement actuel ?

Très insatisfait 🞏 Insatisfait 🞏 Moyennement 🞏 Satisfait 🞏 Très satisfait 🞏

**QUESTION 2 :**

A/ Est-ce que deux comprimés à prendre en même temps à la place d’un seul vous a-t-il ennuyé ?

Pas du tout 🞏 Un peu 🞏 Moyennement 🞏 Beaucoup 🞏

B/ Est-ce qu’avoir deux boites de médicament à la place d’une seule vous a-t-il ennuyé ?

Pas du tout 🞏 Un peu 🞏 Moyennement 🞏 Beaucoup 🞏

**QUESTION 3 :**

A/ Au cours du mois, avez-vous déjà oublié de prendre votre médicament ?

Jamais 🞏 Une fois par mois 🞏 Une fois par semaine 🞏 Plusieurs fois par semaine 🞏

B/ Avez-vous oublié de prendre un des deux comprimés ?

Jamais 🞏 Une fois par mois 🞏 Une fois par semaine 🞏 Plusieurs fois par semaine 🞏

**QUESTION 4 :**

Avez-vous ressenti des effets indésirables ? Oui 🞏 Non 🞏

Si oui, lesquels ……………………………………………………………………………

**QUESTION 5 :**

Souhaitez-vous revenir à votre ancien traitement ?

A/ OUI (tout de suite) 🞏

B/ Peut-être (plus tard, je dois réfléchir…) 🞏

D/ NON 🞏

Si oui, pourquoi ? ……………………………………………………………………………

**EFFICACITE :** Variation de la charge virale ? Oui 🞏 Non 🞏


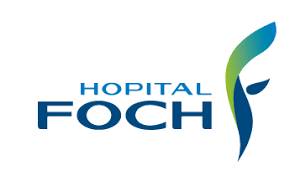
Survey on post ARV de-simplification follow-up

Medical consultation’s date:

Physician: DZ🞏 CM🞏 EF 🞏

| Patient |
| --- |
| Initial of surname : Initial of first name :  Gender : Male / Female Year of Birth :  How long ago did you start the new treatment? ……………………………………………. |

**QUESTION 1 :**

Are you satisfied with your current treatment? Very Dissatisfied 🞏 Dissatisfied 🞏 Moderately 🞏 Satisfied 🞏 Very Satisfied 🞏

**QUESTION 2 :**

A/ Were you bothered by having two tablets to be taken at the same time instead of one?

Not at all 🞏 A little 🞏 Moderately 🞏 A lot 🞏

B/ Were you bothered by having two boxes of medicine per month instead of one?

Not at all 🞏 A little 🞏 Moderately 🞏 A lot 🞏

**QUESTION 3 :**

A/ Have you ever forgotten to take your medication in the previous month?

Never 🞏 Once a month 🞏 Once a week🞏 Several times a week 🞏

B/ Have you ever forgotten to take only one of the two tablets?

Never 🞏 Once a month 🞏 Once a week🞏 Several times a week 🞏

**QUESTION 4 :**

Have you experienced any side effects? Yes 🞏 No 🞏

If yes, which ones ?……………………………………………………………………………

**QUESTION 5 :**

Would you like to go back to your old treatment? A/ Yes (right away) 🞏

B/ Maybe (Later, I have to think about it) 🞏

C/ No 🞏

If yes, why? ………………………………………………………………………………….

**EFFICIENCY:** Change in viral load? Yes 🞏 No 🞏
